# ‘I am always crying on the inside’: a qualitative study on the implications of infertility on women’s lives in urban Gambia

**DOI:** 10.1186/s12978-018-0596-2

**Published:** 2018-09-12

**Authors:** Susan Dierickx, Ladan Rahbari, Chia Longman, Fatou Jaiteh, Gily Coene

**Affiliations:** 1 Centre of Expertise on Gender, Diversity and Intersectionality (RHEA), Vrije Universiteit Brussel, Pleinlaan 2, 1050 Elsene, Belgium; 2 Centre for Research on Culture and Gender, Ghent University, Rozier 44, 9000 Ghent, Belgium; 3 Medical Anthropology Unit, Department of Public Health, Institute of Tropical Medicine, Nationalestraat 155, 2000 Antwerp, Belgium; 4 Amsterdam Institute of Social Science Research, Nieuwe Achtergracht 166, 1018 Amsterdam, The Netherlands; 5 Medical Research Council Unit The Gambia at the London School of Hygiene and Tropical Medicine, Fajara, The Gambia

**Keywords:** Childlessness, Class, Gender, Infertility, Intersectionality, The Gambia, Social suffering, Stigma

## Abstract

**Background:**

There is an increasing awareness that infertility in Sub-Saharan Africa constitutes a severe social and public health problem. Few of the existing studies on infertility explicitly take into account the differences between women. However, how women experience infertility is formed by their various social positions. This research explores the implications of infertility on women’s lives in urban Gambia and aims to provide an in-depth understanding of how this relates to gender and cultural norms as well as different social positions.

**Methods:**

Qualitative data were collected through interviews (33), group discussions (13), participatory observations (14) and informal conversations (31). Purposive and snowball sampling techniques were used to identify participants. The data was analysed thematically using NVivo 11.

**Results:**

Results showed that there was strong social pressure on urban women in The Gambia to procreate. Unable to conform to their gender role, women with infertility were confronted with financial problems, social stigma, as well as emotional and physical violence in their marriage. All women expressed feelings of trauma, stress and sadness. The intersectional approach used in this study highlighted how different positions influenced women’s experiences of infertility. Urban women with a high socio-economic status had a more powerful position within their marriages and the broader community, due to their financial position, professional career and, sometimes, their educational background. In contrast, women from a lower socio-economic background were more likely to be harshly confronted with the social stigma of infertility.

**Conclusion:**

The lives of most women with infertility in The Gambia are characterized by social suffering resulting from gender and pro-natal norms, cultural beliefs and moral concerns, cultural practices and limited access to health care. An intersectional approach is an effective tool to inform public health and social policy since it highlights how, in specific situations, certain groups are more vulnerable than others.

## Plain English summary

In this study infertility is understood to be a condition where women are not able to bear children or are unable to have children following previous pregnancies.

This research explores the impact of infertility on women’s lives in urban Gambia and aims to investigate its relation to the cultural rules of appropriate behaviour for men and women. It will take into account how the impact of infertility differs depending on women’s personal characteristics such as their social class, educational background and religion.

Results show that there is a strong pressure from the community on urban women in The Gambia to have (many) children. Since women with infertility are unable to comply with these expectations, they face financial difficulties. Infertility also influences peoples relationships with their family-in-law, and women with infertility are also confronted with emotional and physical violence in their marriage. In addition, community members confront them with a negative attitude. All interviewed women expressed feelings of trauma, stress and sadness. The background characteristics of urban women influences the ways infertility impacts their lives. Urban women who are financially strong have a more powerful position within their marriages and the broader community, due to their financial position, professional career and, sometimes, their educational background. In contrast, poor women are more likely to be harshly confronted with a negative attitude from community members due to their infertility. Muslim respondents feared that their husband would take a co-wife.

## Background

Infertility is a global reproductive health problem; the World Health Organization (WHO) estimates that 48.5 million couples worldwide are unable to conceive [1]. In Sub-Saharan Africa (SSA) many people are confronted with primary infertility (i.e. when a woman is unable to ever bear a child, either due to the inability to become pregnant or the inability to carry a pregnancy to a live birth) (1.9%) and there continues to be a high rate of secondary infertility (i.e. when a woman is unable to bear a child, either due to the inability to become pregnant or the inability to carry a pregnancy to a live birth following either a previous pregnancy or a previous ability to carry a pregnancy to a live birth) (11.6%). The very high primary and secondary infertility rates in West Africa largely result from poorly managed or untreated reproductive tract infections, including sexual transmitted infections and pregnancy-related sepsis (i.e. postpartum, post-abortion and iatrogenic infections) [2, 3]. Across SSA, access to biomedical health services directed at preventing and treating infertility remains very limited, although this is slowly changing in some countries [4]. The limited services can partly be explained by the relative absence of the topic of infertility on the international health agenda and the confined attention from national policymakers [2, 5–7]. As Harcourt [5] argues, the focus remains on controlling the population growth obscuring the rights of non-reproductive bodies in SSA.

Infertility also constitutes a social problem negatively affecting people around the world [8–11]. Previous anthropological and sociological research has indicated that, due to strong cultural norms to bear (multiple) children, women in SSA countries are often more negatively affected [7, 12–24]. In SSA societies with high levels of gender inequality, regardless of diagnosis, women are blamed for failing to bear children [2, 7, 12, 14, 20, 25–27]. Women may suffer from stigma, social isolation and ridicule within their communities [7]. Fertility problems are also a potential source of tension between partners [13, 17, 27–29]. When a marriage remains childless, some men abuse their wives, verbally or even physically [18, 29]. Women expressed feelings of anxiety, frustration, grief, lack of self-esteem and a general sense of powerlessness [17, 19, 30].

Most of the research on infertility in SSA has been focussed on the impact of infertility on the lives of rural women, and to a lesser extent on men’s experiences of infertility [3, 7, 12, 16]. There is a limited amount of research on the implications of infertility for women living in urban areas [31]. Moreover, few studies on infertility in SSA explicitly consider the differences between women, seemingly assuming the homogeneity of women’s lives and experiences concerning infertility [32]. However, women living in urban areas are a very heterogeneous group, belonging to different social categories (e.g. religious background, ethnicity, class and education level) [33, 34]. The implications of infertility may differ tremendously between women depending on e.g. their class, age, religion, ethnicity [35]. The concept of intersectionality provides an analytical tool to grasp the multifaceted realities of women’s lives which co-exist and interact [35–38]. It allows us to understand how social categories influence the lives of women with infertility and how the intersection of these social categories may lead to different and distinct experiences of oppression and opportunity. It looks into the context-specific interactions of these social categories and the power relations underpinning inequalities.

The existing research on infertility in SSA is overwhelmingly conducted in South and Central Africa, with less attention paid to infertility in West Africa [14, 39]. Given the political, economic, cultural and social differences between these regions, there is a need to understand the implications of infertility in West Africa. This paucity of research on infertility in West Africa is also reflected in the few studies conducted in The Gambia [2, 15]. The Gambia has a score of 0.641 on the gender inequality index indicating high levels of gender inequality [40–45]. The total fertility rate remains high in The Gambia with 5.8 live births per woman [46]. The studies reporting infertility rates in The Gambia are quite old, with a contraceptive prevalence study conducted in 1993 stating that around 2% of women at the end of their reproductive period had no children [47]. Considerably more women (11.6–15.2%) had a longer than expected time interval since their last birth. Another study found 9.8% of participants to be infertile, with secondary infertility (i.e. no pregnancy after at least 12 months of regular unprotected sexual intercourse) affecting 8.8% of all participants [24].

This qualitative study provides an in-depth understanding of the implications of infertility on women’s lives in the urban communities of the West Coast region of The Gambia today and how these relate to broader gender norms and cultural practices. In order to understand the complexities of the lives of women with infertility, the analysis relies on the conceptual framework of intersectionality.

## Methods

### Study setting

Fieldwork was conducted between September 2017 and April 2018 in the urbanized West Coast region of The Gambia (e.g. Bakau, Brufut, Banjul, Fajikunda, Old Jeshwang, New Jeshwang). The Mandinka are the largest ethnic group in The Gambia, comprising 42% of the population, followed by the Fula (18%), the Wolof (16%), the Jola (10%) and the Serahule (9%) [48]. The pre-dominant religion in The Gambia is Islam (90%), 8% are Christian and 2% other. Polygyny is a common practice among Muslims; a study reported that 54.3% of married women live in a household with one or more co-wives [24].

### Research design

This research is part of a larger anthropological research project on gender, culture and infertility in The Gambia (West Coast region) and Senegal (Casamance). This research opted for a qualitative research approach, combining interviews, group discussions and participant observation with informal conversation. Method triangulation was used to provide an in-depth understanding of the research topic and to enable the researchers to reflect on local narratives and personal stories of the participants. When respondents were fluent in English, the interview took place in English. However, in most cases conversations were carried out with the assistance of a trained translator in Mandinka or Wolof. It is unavoidable that some level of meaning was lost in translation and transcription, as personal perspectives influence the interpretation and translation process [49]. To minimize this, the translator was trained extensively for four days. The translator was familiarized with the themes and supplied with a question guide with potential questions that were translated beforehand. This allowed the first author and translator to discuss the terminology, phrasing and translation of potential questions. During data collection, attention was paid to non-verbal communication such as body language, gestures and silences.

### Interviews

A total of 33 semi-structured interviews were conducted by the first author at locations where respondents felt at ease (Table 1). Most interviews were recorded but if the interviewee preferred not to be recorded or the interviewer was under the impression that it might make the respondent uncomfortable, the responses were written down in detail during or after the interview. Interviews lasted approximately one hour. The interviews were conducted with a flexible question guide. The question guide started by capturing the basic socio-demographic information, this was to help respondents feel more at ease. Then open questions were asked concerning (i) people’s personal history and marriage, (ii) the impact of infertility, (iii) the relationships with partner, in-laws and community members and (iv) emotional consequences.

**Table 1:** Overview of the interviewed respondents’ socio-demographic characteristics

Interview	Education level	Main source of income	Ethnicity	Religion	Marital status	Age category^a^
1	University Gambia	Housewife	Mandinka	Muslim	Monogamous marriage	Adult
2	University abroad	Civil servant	Mandinka	Muslim	Monogamous marriage	Adult
3	University abroad	Civil servant	Mandinka	Muslim	Monogamous marriage	Adult
4	University abroad	Civil servant	Mandinka	Muslim	Monogamous marriage	Adult
5	University abroad	Civil servant	Mandinka	Muslim	Monogamous marriage	Adult
6	No education	Market vendor	Mandinka	Muslim	Polygynous marriage	Elder
7	No education	Housewife	Mandinka	Muslim	Divorced	Adult
8	University abroad	Civil servant	Mandinka	Muslim	Monogamous marriage	Adult
9	No education	Market vendor	Mandinka	Muslim	Widow	Elder
10	No education	Housewife	Fula	Muslim	Widow	Elder
11	No education	Cleaning lady	Mandinka	Muslim	Polygynous marriage	Elder
12	No education	Market vendor	Masuwanka	Muslim	Polygynous marriage	Elder
13	No education	Market vendor	Mandinka	Muslim	Polygynous marriage	Elder
14	No education	Farming	Fula	Muslim	Polygynous marriage	Adult
15	No education	Market vendor	Wolof	Muslim	Polygynous marriage	Adult
16	High school	Housewife	Wolof	Muslim	Monogamous marriage	Elder
17	No education	Market vendor	Mandinka	Muslim	Polygynous marriage	Elder
18	No education	Housewife	Mandinka	Muslim	Polygynous marriage	Adult
19	No education	Market vendor	Mandinka	Muslim	Polygynous marriage	Elder
20	No education	Housewife	Serer	Muslim	Polygynous marriage	Elder
21	Arabic school	Market vendor	Mandinka	Muslim	Polygynous marriage	Elder
22	No education	Housewife	Mandinka	Muslim	Polygynous marriage	Adult
23	Arabic school	Housewife	Mandinka	Muslim	Polygynous marriage	Adult
24	University abroad	Civil servant	Mandinka	Muslim	Monogamous marriage	Adult
25	University abroad	Civil servant	Aku	Muslim	Monogamous marriage	Adult
26	University abroad	Civil servant	Aku	Muslim	Monogamous marriage	Adult
27	University abroad	Civil servant	Aku	Muslim	Monogamous marriage	Adult
28	No education	Gardening	Mandinka	Muslim	Polygynous marriage	Elder
29	High school	Housewife	Karoninka	Muslim	Monogamous marriage	Adult
30	University Gambia	Housewife	Mandinka	Muslim	Polygynous marriage	Adult
31	College	Teacher	Aku	Christian	Monogamous marriage	Adult
32	Arabic school	Business woman	Mandinka	Muslim	Monogamous marriage	Adult
33	Arabic school	Cleaning help	Mandinka	Muslim	Monogamous marriage	Adult

^a^Adult: 18–49 years old/Elder: + 50 years old

### Group discussions

In total, 13 group discussions were conducted either when informants wanted to be interviewed together, or when the researcher wanted to collect different views on a particular topic in which case a group interview was requested. The number of participants varied between three up to nine people. Two group discussions on the topic of divorce and the position of women with infertility in urban Gambia include both male and female respondents. The other eleven group discussions on aetiological beliefs and the position of women with infertility in Gambian society were conducted with women experiencing infertility. Group discussions took place in several places including the privacy of people’s homes, the offices where people were working and public spaces such as restaurants.

### Participant observations

Participant observation consisted of participating in everyday activities, such as cooking and cleaning in the compound. The main author also went to the Muslim Cadi Court, attended naming ceremonies and participated in a march to increase awareness about infertility. Notes were taken whenever possible and a voice recorder was used to record findings when the situation did not allow to take notes. These observations offered the opportunity for 31 informal conversations on relevant themes with community members and women experiencing infertility. Participant observation was important for the research project, providing a more contextualised understanding of the research questions. This method also facilitated gaining access to respondents and building trust between the researcher and participants. This was particularly important given the sensitive nature of the research topic and questions, allowing to minimize socially desirable or otherwise biased answers.

### Sampling framework and method

When analysing infertility, it is important to acknowledge that standard epidemiological and demographical conceptualizations of infertility, subfertility, miscarriage and stillbirth are not always meaningful for people living in low- and middle-income countries [19, 21, 50–53]. Conceptualizations about what is natural, normal or expected in terms of fertility varies in time and over situations [18, 26, 53]. Therefore, in this study women were defined as infertile when they considered themselves as such, regardless of the duration of their fertility problems and their number of living children. In practice this meant that in our study sample, people were included who did not have children or who already had children. Purposive and snowball sampling strategies were utilized to enable maximum variation of identified participants through gradual selection processes. Critical cases (i.e. informants that presented us with important or unusual findings) were continuously included in the sample frame. These sampling techniques enhanced participants’ trust and confidence in the researcher. When possible, informants were visited multiple times to increase levels of familiarity and trust between researcher and respondents to reduce bias.

### Data analysis

The process of analysis began whilst still in the field, this included both process and thematic content analysis. Process analysis included self-reflectivity and discussion with the translator on methodological approaches. Thematic data analysis was an explorative, concurrent and iterative process including several stages [54]. First, incoming raw data was analysed to inform further data collection. Second, each incoming transcript was read and open codes were generated in NVivo 11 Analysis Software (QSR International Pty Ltd. Cardigan UK) (e.g. experiences with polygyny, importance of children). The third phase consisted of axial coding to create themes by grouping open codes according to their coherence after careful reading the analyses of the coded text fragments with attention to potential interrelations (examples of themes are pro-natal norm, the economic impact of infertility, the emotional impact of infertility). This served as the basis for a final code tree design. Throughout the analyses data were constantly set against findings of the existing literature. The first author received feedback from the co-authors based on analytic discussions, which informed the final interpretations. The emergent codes and themes and the relevance of women’s social positions are discussed below, supported by respondents’ quotes. Specific quotes were mainly chosen from interviews based on their intrinsic richness on a particular theme.

### Positionality and reflexivity

The first author had resided in The Gambia prior to carrying out this research (in total 14 months over the course of three years). She was informally adopted by a local family and acquainted with culturally appropriate expressions and behaviour. Her position as a married woman was important in facilitating discussion around to marriage and fertility. Personal stories were shared by both the researchers and the participants during interviews, aiming to make the interview more intimate and reciprocal [55]. The interviewer’s positionality – as a white, Western woman – made her at times an outsider which might have influenced the answers provided by the respondents [49, 56]. This twofold position as insider and outsider was helpful for some of the respondents, as it made the researcher a confidential conversation partner for those who wanted to share their stories and secrets. The emotions shared during the research made the lead researcher reflect on ethical questions of moral responsibility, injustice and powerlessness.

### Ethics, consent and permission

Ethical approval for this study was received from The Gambia Government/MRC Joint Ethics Committee (SCC1562) and the ethical commission of the Vrije Universiteit Brussel (Belgium). The interviewer followed the Code of Ethics of the American Anthropological Association (AAA). People were informed about project goals, the topic and type of questions as well as their right to decline participation or to interrupt the conversation at any time. Anonymity was guaranteed and confidentiality assured. If people consented to be interviewed, during each interview participants were asked if they had any questions.

## Results

### Positionality of respondents

Throughout the presentation of the results, references are made to the influence of different social positions of women with infertility and the impact of relevant cultural practices where relevant. This makes it important to indicate the socio-demographic characteristics of the respondents, and how these interrelate. While some respondents of the informal conversations, interviews and group discussions were illiterate and in a more vulnerable financial position living from subsistence farming, others were highly educated with a secure job in the public sector or owned a business. In this study sample, the educational background of respondents related to women’s professional career – in particular women who were educated abroad had a good job. However, there was no straightforward causality: one uneducated woman did run a successful business, while other well-educated women were stay-at-home mothers with no personal income. People born and educated in rural areas were more likely to be financially vulnerable. They also tended to have married quite young in an arranged marriage to a distant relative. In contrast, families who had been living in the urban area for several generations were more likely to have gone to school, have a stronger financial position and were less likely to be in an arranged marriage. In this study sample, people’s educational and financial background was unrelated to their ethnic and religious background.

### Pro-natal norm

The data showed that in order to understand the impact of infertility on women’s lives, it is important to describe the pro-natal norm in Gambian society. All respondents of group discussions and interviews said married people should become parents. Observations and group discussions showed that there is a strong orientation in urban Gambia towards family life and values. An important reason given for having male children in this patrilineal society is the continuation of the family lineage. Observations indicated that the family unit was held together by complementary gender and age roles; women were responsible for the running of the household and men should be the financial providers. Considerations about the future welfare of the family were also relevant to understand the pro-natal norm. Older respondents explained that in the absence of a welfare system, more hands to work meant more revenue for the family, leading to more savings and security. In contrast, respondents who went to school expressed that they did not want to have more than four children since this implied a financial burden for the future well-being of their family. Interviewees also wanted to have some daughters because they were considered trust worthier to take care of their parents during old age. Women with infertility also explained that children are an important source of fun, pride and companionship.

### Gender aspect of infertility

Married couples that remained childless were not in accordance with dominant norms prescribing them to have children. Respondents from group discussions and interviews agreed that the perceived cause of infertility is gendered. Regardless of any (biomedical) diagnosis, women are often perceived to have fertility problems. While within their communities and families most interviewed women seemed to accept their responsibility for not having children, during private interviews several interviewed women confided they found it troublesome that women were always accused of infertility.

‘*Some community members would say “she takes family planning tablets which destroys her stomach that is why she could not have children”, some will say “the woman has jinnee [spirits] that is why she could not have children”, but their minds would never tell them that because of the husband she cannot have children.’* (Interview 7)

### The economic impact of infertility

*‘The economic impact is that you tend to spend a lot. Like me, I have spent a lot, a lot of my savings, three quarter of my savings, going to doctors, doing different tests. I do research online and buy supplements that you can use to boost your fertility. Whatever you name it, I have tried.’* (Interview 2)

Regardless of women’s financial position, infertility has an economic impact on their daily lives. Muslim women without children were in vulnerable positions when their husband died. Observations at the court and interviews indicated that local interpretation of Sharia law was often followed closely when it came to inheritance, and sons received at least double the share of daughters and wives. As a result, in-laws or co-wives could expel widows without children from the compound with few resources to rely on. This is illustrated in the story shared by a Muslim adult woman with infertility in a household with a co-wife with sons living in Europe (interview 15). She explained that when her husband died, her co-wife wanted to sell her house. The woman with infertility was desperate that she would need to live on the street and pleaded so that she could stay inside the compound. This was accepted by the co-wife. However, the co-wife still did not want to cook together nor share the electricity. Since the woman had limited resources she used candlelight, until she got money from a women’s savings organisation allowing her to pay her own electricity meter.

The economic position of the couple also has an impact on their health-seeking behaviour. In order to conform to dominant gender roles, initially often both partners – regardless of their financial position – would invest resources in order to find treatment. However, when this treatment-seeking did not lead to children, the financial support of the husband commonly decreased. As this quote from interview 29 indicates:

*‘You know sometimes when men spend a lot looking for health care and they didn’t see any result, they will sometimes feel reluctant to pull out more money.’*

Most respondents explained that they continued to look for treatment even when their husband no longer provided financial support. Observations at the public and private clinics and interviews indicated that for women who had a salaried job, it was much easier to look for treatment independently from their husband. Not only the financial position of both partners had an impact on people’s treatment seeking, education was also relevant. Educated women were more likely to look for biomedical health care:

*‘There is a lack of education and sensitization. Some of the uneducated women, you do not blame them because they do not know that they have to go and see the medical doctor. They do not know that infertility is not about marabouts [i.e. traditional healers].’* (Interview 2)

Observations and interviews indicated that only women with infertility who were financially strong could afford to travel abroad for medical treatments that are not available in The Gambia.

Lastly, infertility also had an impact on women’s daily livelihood. While our research indicated that gender roles prescribed that men should provide ‘fish money’ (i.e. money for the daily livelihood of the wife), in practice many women with infertility complained they did not receive fish money. This was particularly stressful for women who did not have a salaried job as an educated woman explained during a group discussion:

*‘Having children is also important for the economic security of women. When a woman remains childless, the husband will refuse to give his wife fish money or to provide clothes. She is chased away directly, but commonly indirectly by not providing financial support. These women will often return to the compound of their parents.’*

### The impact of infertility on social relationships

#### Differential social experiences depending on women’s position

Most interviewed women experienced difficulties within their social relationships because of infertility. However, the impact of infertility on women’s lives differed to some extent depending on their financial position. The interviewed women in strong financial positions often had more power to negotiate their relationships both with other community members, their family-in-law and within their marriages. Some of them supported their family-in-law financially. Their economic strength implied they could take care of themselves even when divorced. Therefore, they were less afraid that their husband would abandon them or remarry. The story told by an educated woman with infertility illustrates this: *‘My husband’s brother divorced from his wife after ten years of marriage because they could not have a child. The lady decided to break the marriage due to the social stigma and the in-laws. She has her work, she is educated and has her papers so why allow people to traumatize you?’* (Interview 5). Furthermore, women with a good job had the opportunity to go outside of their community to work, which allowed them to escape from the pressure to become pregnant.

Despite their stronger financial and social position, women with a salaried job also explained that they were confronted with stigma and occasionally community members would accuse them of not wanting to have children. As these women described themselves as *‘desperate to have children’*, these comments were experienced as painful and resulting from ignorance.

In contrast, interviewees who didn’t have a well-paid job were more likely to be restricted to their traditional roles as daughters, wives and mothers and have less opportunity to escape the tensions and hurtful comments from their family-in-law and other community members.

*‘Sometimes people can even be saying words to you that hurt you. There is a lot of social stigma. I got married and I had my first child after five years of marriage and that one passed away […] It took me another four years to become pregnant. Sometimes I will be sitting down, I will be hearing comments, they will be throwing words at me, but you know: people are different, we are the educated ones. We have our work and everything, you can’t traumatize us, but what of the poor women in the household?’* (Interview 5)

#### Stigmatization within the community

All interviewed women with infertility explained that gossip, jokes and rumours about infertility spread easily within communities. Women corroborated about the suffering caused by community members, being called ‘*barren’*, *‘a witch’* and ‘*eating their own children’*. This social stigmatization can be partly explained by (i) gender norms and roles; and (ii) the perceived aetiology of female infertility. While respondents discussed several aetiological interpretations during interviews and group discussion, two of these are relevant for this discussion. First, infertility can be associated with witchcraft whereby women were accused of being witches eating their own children. Second, while premarital and extra-marital intercourse are considered immoral, infertility was associated with abortions, sexual infections and sometimes ‘overdoses’ of family planning injections and tablets. Despite the stigmatization of women with infertility, few of our respondents were explicitly excluded from attending social events such as naming ceremonies and weddings. When it did happen, it led to alienation, as explained during an interview:

‘*Yes, I used to feel shy. Some will say about me: “a woman who refuses to give birth is coming”. Even if I want to eat porridge, they will say “do not touch this porridge, it is only for women who gave birth”, and if there is naming ceremony, they would not inform me’*. (Interview 9)

#### Adverse consequences for relationship with in-laws

Observations, group discussions and interviews indicated that two cultural practices increased the tensions in the relationship between women with infertility and their family-in-law. Firstly, the virilocal residence pattern, whereby the newly-wed moves in to her new marital family home. In this new home, she has to negotiate her position with her family-in-law, and having a child helped in this process. Respondents who did not live with their extended family were on both extremes of the socio-economic classes: some originated from rural areas and were renting a small house, unable to afford their own place, or they came from a high socio-economic class and could afford their own house separated from the family-in-law. Several respondents who stayed in the compound with their family-in-law were confronted with harassment and pressure by the in-laws, in particular the mother-in-law. Secondly, the bride price increased the pressure on women. Bride prices illustrated the exchange between families: the transfer or reproductive and productive capacities of a woman to her husband’s family. This bride price differed between ethnic groups and families, and had to be discussed among the families involved, but it often consisted of large amounts of goods, cash or animals. An argument often made by the family-in-law is that despite the bride price, they did not get a ‘good’ wife in return. They encouraged the husband to marry another wife as the excerpt of interview 9 indicates:

Respondent: ‘*My [previous] husband’s family told my husband to divorce, or get a second wife, by then his mother and sisters where all living with me. I used to quarrel with them.’*

Interviewer: ‘How did your husband deal with that pressure from the family?’

Respondent: ‘*My [previous] husband loves me so much but he used to tell me that if you do not have a child, I will marry a second wife.’*

#### Adverse consequences for marriage

Regardless of the type of marriage, through time, few couples were able to manage with the burden of infertility and remain supportive of each other. Many respondents suffered because of this. During a march to support couples experiencing infertility, a young woman said: *‘men are very hard. They don’t love you when you are faced with fertility difficulties.’* The long-term impact of infertility on marital relationships was diverse.

Firstly, some women experiencing infertility spontaneously said they were beaten by their husband because of their condition: *‘I sat for so many years without having a child. When I came to bed my husband used to beat me, beat me, beat me! And he said that I am not a woman; that I cannot be pregnant.’* (Interview 19).

Secondly, during interviews women with infertility explained their fears of a divorce. A woman who was first married at the age of 14 to a 50-year-old man explained that during this period she did not even want to be pregnant and ran away several times. However, when she was in her second arranged marriage, she feared her new husband would divorce her when she did not get pregnant within the first four years of her marriage which proved to be true: *‘I said to myself: “if he divorces me, where would I go? And even if I remarry, it [the infertility] is still going to be the same problem”’* (Interview 9).

Thirdly, women expressed that the lack of social and financial support is very painful. As a woman with infertility said: *‘If he neglects you and you go to your people, all they would say is that “your husband loves you, if he didn’t love you he would have divorced you, so go back to your husband’s house and have patience. In the long run he will come back to you”. That patience is very painful. When you feel that stress, it may make you lose your life because stress affects your heart’* (Interview 9)*.* The following extract of interview 11 with a woman who lost seven children either during pregnancy or delivery indicates the suffering due to the lack of support from her husband:

Respondent: ‘*If I lost my baby that is the end of my husband’s support. He will not give me courage or make me active or to feel supported unless I become pregnant again.’*

Interviewer: ‘How did you feel at that moment?’

Respondent: ‘*Death is the will of God but we need to share the sadness together, so it made me feel sad.’*

Fourthly, while none of the respondents talked about their personal situation, they would share gossip about both Christians and Muslims engaging in extramarital relationships. These extramarital relationships clash with gender norms for both men and women. However, in practice, (i) consequences are less severe for men since it was regarded as being in the ‘nature’ of men to have sexual relationships outside of the marriage and (ii) some interviewed women confessed they thought that if the husband was likely to be infertile it was better to become pregnant from a stranger than to divorce.

Lastly, many of the Muslim respondents feared their husbands would engage in polygyny, and 15 of the interviewed women were already in polygynous marriages. While men were supposed to engage in polygyny only if they had the financial means to treat all wives equally, in practice men often did not treat their wives equally leading to tensions between co-wives. When a co-wife was able to have children, for wives experiencing infertility it was a daily reminder of their own inability to have children.

Despite the marital problems many of the interviewees faced, women rarely left their unhappy marriages. Interviewees explained that marriage is an important social and cultural institution. Therefore, most of the divorced and widowed respondents re-married quite soon after losing or divorcing their husband.

### Emotional impact of infertility

*‘Sometimes I face challenges. When things become difficult, sometimes I feel like crying. I would say to myself: “what if I had more than one child? It might be that when it is difficult with one child, the other child might be able to support” […]. For me, my mother and father passed away, and the man I am married to now [her first husband died], he is an old man and financially we are both not strong, so I leave everything in the hands of God’* (Interview 15).

During some interviews, women cried discussing the financial and social problems they were confronted with in their lives. Interviewed women with infertility said they were often under stress (*neku-yaa*, lit. translated as an unsteady mind), which led to heart problems. Respondents experiencing infertility said it was the greatest grief they had in their lives and explained they felt desperate. They reported symptoms of depression ranging from extended periods of crying to isolation. This is further corroborated by the silence surrounding the topic of infertility, complicating women’s ability to talk about their stress and fears. As a woman who lost seven children either during her pregnancy or delivery explained: *‘Most of the time whenever I lost my child, my co-wife didn’t take it as something serious or sympathized with me. That is something that affects me a lot and I feel bad’* (Interview 6)*.* Feelings of hope, sadness and frustration were for many linked to the (sometimes) long periods (up to 20 years) of looking for health care. Expressions of sadness and loss were common among women who had salaried jobs but they explained that they found it helpful to be able to focus on their work.

## Discussion

Although the authors want to warn against the victimisation of women experiencing fertility problems, it remains important to articulate the precarious situation of many of these women in urban Gambia. This study showed that motherhood is still considered an essential aspect of the adult female role, leaving the pro-natal norm unquestioned. What has changed among the urban population is the desired gender and number of children. That is (i) daughters are perceived to be trustworthy – they can be relied on during old age – and (ii) young educated urban women prefer to have less children, which enables them to support their children’s education. Initially it was believed that when couples did not have children, it was the women’s fault. Due to dominant unequal gender norms and roles, women had more social pressure to overcome their condition and become pregnant.

As in other SSA countries [12, 18, 20, 30], the lives of women with infertility in urban Gambia were marked by various kinds of economic, social and emotional problems. The concept of social suffering can be used to describe the impact of these various problems on women’s lives. Social suffering refers to the social misery, pain and hurt people experience individually and collectively due to institutionalized inequalities and systematic discrimination [57, 58]. It is problematic that infertility is seldom recognized as a key reproductive health priority at the international and national level [5, 22, 59]. This lack of commitment stands in stark contrast with the stigmatization of women experiencing infertility in urban Gambia. This social stigma is not only related to the normative construction of women to become mothers, but also to the cultural beliefs and moral concerns about the aetiology of infertility. The social pressure to become pregnant was even more stressful as a result of cultural practices such as the bride price and virilocal residence patterns. All women experiencing infertility feared marital problems and many women reported emotional and financial neglect and abuse.

By employing an intersectional framework, this study attended to the subtle differences between women’s experiences of infertility depending on their social position. An intersectional framework is rarely used within anthropological or health system research in low- and middle-income countries [36, 60, 61]. Although recently, several authors [36, 62] have stressed the advantages of an intersectional approach to understand complex social situations. In anthropological or social science research on infertility, few studies have explicitly taken into account the different social categories influencing women’s lives. In urban Gambia, the manifestation and intensity of the financial, social and emotional problems differed between women with a strong financial position and women with a more vulnerable financial position. In general, the stronger economic position gave these women more resilience to deal with stigma. Being financially independent, they had a more powerful position within their marriage to negotiate relationships with their husbands and in-laws. A salaried job made women more resistant towards outside pressures by providing them an independent space outside the compound and community. Furthermore, women who were financially strong were less dependent on their husbands to look for health care and found it easier to go to private health centres or to travel abroad for IVF treatment.

This study contributes to the few earlier studies looking at the influence of social positions on the lives of women with infertility in low-and middle-income countries. Inhorn [32] was the first to discuss the importance of class differences on the stigma faced by women with infertility in urban Egypt. Women with a lower social status were more devalued, disempowered and stigmatised. Nahar & Richers [34] studied the position of women experiencing infertility in Bangladesh doing research among both poor rural woman and wealthy urban women. The main difference found was that rural childless women had more social pressure. As in these studies, also in urban Gambia, people with fewer financial resources suffer more. However, taking into account their intersectional position, including the relevance of religion, educational background and having a salaried job also influenced these women’s experiences.

## Conclusion

Intersectionality is an effective tool of analysis and for informing social policy since it highlights how in specific situations social positions and identities interact and influence people’s behaviour. Our findings stress the urgent need for targeted services for all individuals and couples experiencing infertility in The Gambia. Infertility has negative financial implications, leads to social stigmatization, marital problems and emotional difficulties. Therefore, the capacity of the health system needs to be built not only to prevent and treat fertility problems among both women and men, but also to follow up and provide psychological and emotional support especially for women. While all women would benefit from these services, special efforts should be made to target those women who have little financial and social resources.

## Abbreviations

SSA: Sub-Saharan Africa
WHO: World Health Organization
